# A 3D U-Net Based on a Vision Transformer for Radar Semantic Segmentation †

**DOI:** 10.3390/s23249630

**Published:** 2023-12-05

**Authors:** Tongrui Zhang, Yunsheng Fan

**Affiliations:** College of Marine Electrical Engineering, Dalian Maritime University, Dalian 116026, China; ztr020917@dlmu.edu.cn

**Keywords:** radar semantic segmentation, data cube, 3D U-Net, transformer

## Abstract

Radar data can be presented in various forms, unlike visible data. In the field of radar target recognition, most current work involves point cloud data due to computing limitations, but this form of data lacks useful information. This paper proposes a semantic segmentation network to process high-dimensional data and enable automatic radar target recognition. Rather than relying on point cloud data, which is common in current radar automatic target recognition algorithms, the paper suggests using a radar heat map of high-dimensional data to increase the efficiency of radar data use. The radar heat map provides more complete information than point cloud data, leading to more accurate classification results. Additionally, this paper proposes a dimension collapse module based on a vision transformer for feature extraction between two modules with dimension differences during dimension changes in high-dimensional data. This module is easily extendable to other networks with high-dimensional data collapse requirements. The network’s performance is verified using a real radar dataset, showing that the radar semantic segmentation network based on a vision transformer has better performance and fewer parameters compared to segmentation networks that use other dimensional collapse methods.

## 1. Introduction

In recent years, there has been significant progress in the field of optical image processing [1,2,3]. However, the quality and effectiveness of optical images can be impacted by weather conditions and the passage of time [4]. Radar technology, on the other hand, uses electromagnetic waves at lower frequencies than visible light, making it have stronger robustness and much more adaptable to different environments, and enabling detection over much greater distances [5]. As a result, radar has found a wide range of applications in fields such as robotics, autonomous driving [6], missile guidance [7], remote sensing [8], and underground exploration [9]. By focusing on radar semantic segmentation, researchers can effectively improve the accuracy and automation of radar target identification, which in turn can help advance the development of different fields. It should be noted that this paper is an extension to a previous conference paper [10].

Traditional methods for automatic target recognition in radar are typically based on radar cross-sectional area, radial profile, or modulation information. However, with the advancement of deep learning technology, researchers have started to explore the use of deep learning algorithms for radar target recognition [11]. The study of radar semantic segmentation can play a critical role in enhancing the accuracy and automation of radar target recognition. Specifically, research in this area can lead to significant improvements in both the accuracy and degree of automation of radar target recognition.

Due to the directionality of radar signals, different orientations and angles can produce distinct radar semantic segmentation results by processing the original echo signal from different directions. Zhang Haoyu and colleagues were able to detect radar jamming by utilizing the complex-valued Analog-to-Digital Converter (ADC) [12] and fully extracting the inherent characteristics of radar jamming signals. This method has demonstrated improved recognition accuracy compared to the use of Valued-ADC. More importantly, the research proves the great potential of image processing algorithms in the radar recognition field, which opens up a new way for radar target recognition. Shao Guangqing et al. proposed the use of a siamese network based on Convolutional Neural Networks (CNN) to address the challenge of limited radar training samples for radar jamming signal classification [13]. It shows that CNN has good performance in radar interference recognition.

Many researchers aim to reduce computation by using data obtained after ADC processing for target recognition. Zhaoxi Chen et al. implemented personnel recognition and gait classification using time-frequency spectrograms in their study [14]. They utilized directional diversity offered by multistatic radar to improve classification accuracy, and their experimental results were superior to other methods. Wang Yafeng et al. used CNN to process time-frequency spectrograms for radar active-jamming recognition [15], proving that convolutional neural networks possess strong capability in distinguishing active jamming and can prepare sufficiently for anti-interference processes. Chen Xiaolong et al. combined time-frequency spectrograms with ADC data for target detection, proposing a two-channel CNN and a false-alarm-controllable classifier-based marine target detection method from a feature extraction and classification perspective [16]. However, radar data’s particularity means that the above data still occupies a large number of computing resources. Sun Yuliang et al. extracted the N points with the highest intensity in a radar heatmap, encoding the point cloud information of these points into a feature cube as input for the model, to improve the robustness of radar-based identification systems [17]. Because it only uses N points as input, their work has fairly low latency.

In recent years, the widespread adoption of radar heatmaps for semantic segmentation has been observed due to the improvement of computing power. Sun Yuliang et al. proposed a network architecture that employs a two-dimensional radar heatmap input with three channels representing different attributes. These attributes include pitch angles, azimuths, and amplitudes of varying speeds at different times, respectively [18]. This innovative approach reduces data volume and enhances the network’s ability to process the information contained within the radar heatmap, ultimately improving its overall performance. Another network architecture designed by Martinez et al. introduced prior information into the model by taking into account the attributes of radar images, which significantly improved the recognition performance of radar heatmaps [19]. Sangtae Kim et al. incorporated the long short-term memory network into radar signal processing and devised a deep neural network comprised of convolutional recursive units to enhance the dynamic target recognition capability in automotive radar systems [20]. This innovation has significantly improved the accuracy of target classification.

The aforementioned researchers utilized 2D radar heatmaps, which typically contain only two-dimensional spatial information similar to visible images. However, to improve recognition accuracy, 3D radar heatmaps are now widely used for radar target recognition. Unlike 2D radar heatmaps, 3D radar heatmaps can contain depth or velocity information in addition to 2D spatial information. Due to its cube-like appearance, the 3D heatmap is often referred to as a data cube. Compared to other radar data forms, the data cube provides almost complete, low-redundancy information with a moderate amount of data. Bence Major et al. made a groundbreaking discovery when they employed radar heatmaps to detect road targets, marking the first time this technique was used [21]. By collapsing a dimension of the data cube to obtain three-channel input, they successfully achieved road target detection. In another study, Andras Palffy et al. utilized a technique of cutting the radar cube around the target as input, in order to reduce computational requirements [22]. This radar cube, containing the local speed information of the target, significantly improved the accuracy of classifying road targets. Building on this concept, Gao Xiangyu et al. developed a new multi-angle radar convolutional neural network by slicing the data cube, which resulted in even better accuracy for semantic recognition of road targets, showing potential for low-cost radar as a viable alternative to optical sensing under harsh conditions [23]. However, the models mentioned above all utilized collapsed or incomplete data cubes as input for recognition, leading to a significant loss of information, such as the velocity axis being cropped. This missing information could contain crucial and representative data, as seen in Doppler Radar where the propeller speed intensity of an aircraft may have significant characteristics despite being low.

**Figure 1:**
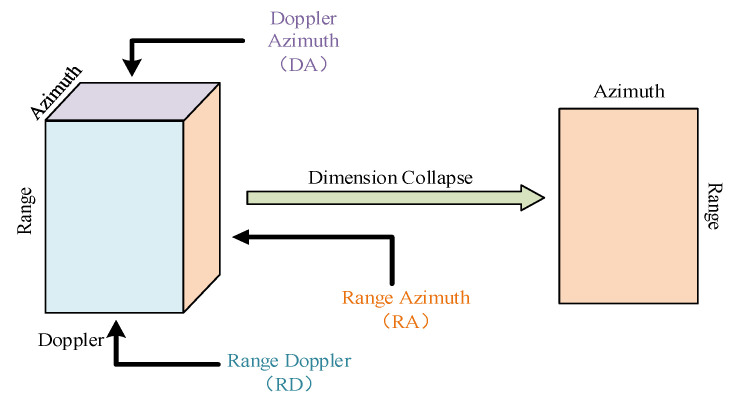
3D data in the encoder is collapsed into 2D data.

This paper proposes a novel approach to address the issue of data compression in the data cube, by introducing a complete data cube-based radar semantic segmentation network called 3D Collapse U-Net (3DCUNet), which significantly improves the accuracy of radar target recognition. The study builds upon the 2D U-Net architecture and extends it to 3D data processing. Unlike traditional U-Net, the encoder and decoder of 3DCUNet have different data dimensions, and to address the problem of inconsistent dimensions between inputs and outputs, the 3D data in the encoder needs to be collapsed into 2D data as shown in Figure 1. However, collapsing directly through the pooling operator can result in the loss of feature information. To overcome this, this paper presents a novel dimension collapse module based on the vision transformer. By leveraging the vision transformer’s ability to extract global features, this module can collapse 3D data into 2D data and reduce effective information loss. This results in the adaptive characterization of input data and effectively narrows the domain differences between the encoder and the decoder. Finally, the paper proposes a network that incorporates the dimension collapse capability for efficiently processing the radar data cube and performing radar semantic segmentation. In summary, the paper makes two significant contributions:

1. We utilize a Vision Transformer to collapse 3D data into 2D data, offering a more efficient method for processing high-dimensional data, which has the potential to be applied to other cases of high-dimensional data collapse, making it a versatile and valuable tool in data processing.
2. In this paper, we propose a Radar Semantic Segmentation Network, 3DCUNet-based and reliant on complete data cubes, which achieves automatic recognition of radar targets and advances the application of radar heatmaps.

The remainder of this article is structured as follows: Section 2 provides a comprehensive introduction to the Radar Semantic Segmentation Network. Section 3 demonstrates the experimental results and validates the algorithm’s efficiency. In Section 4, we discuss the experimental results in detail. Finally, Section 5 summarizes the conclusions of this paper.

## 2. Methods

**Figure 2:**
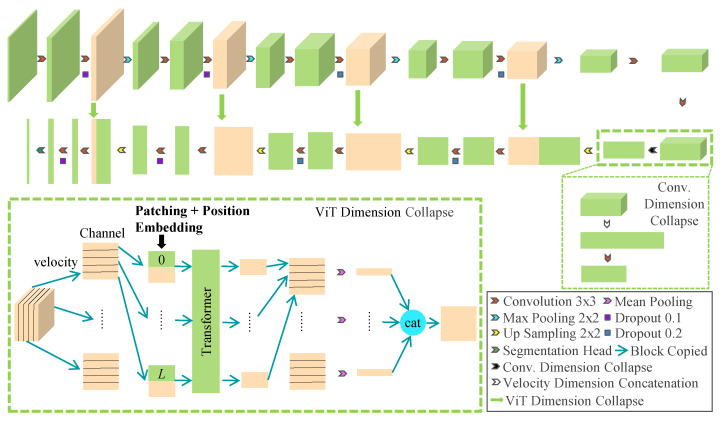
The complete network architecture of 3D Collapse U-Net.

The format of data used in this work is depicted in Figure 1, which differs from the conventional data format in that it consists of three dimensions: range, azimuth, and velocity. Considering one channel as a dimension, it becomes four dimensions. The velocity dimension comprises multiple parts with different speeds of the same target. Therefore, there is no need to divide the velocity dimensions belonging to the same target in the output segmentation results, resulting in only two dimensions of range and azimuth. For radar data with this data format, a new radar semantic segmentation network is proposed, called 3D Collapse U-Net, which comprises encoders and decoders, as depicted in Figure 2. The encoder phase employs 3D Convolution (Conv.) to extract the features of input range-azimuth-velocity data, better characterizing the radar target. The final segmentation result, being two-dimensional, is generated using 2D Conv. in the decoder phase. Obviously, the encoders and decoders in the 3D Collapse U-Net model have different data dimensions, which can create challenges for accurate information processing. Previous studies such as [22,23] have addressed this issue by employing a fixed clipping algorithm to input high-dimensional data into the model. Although this method reduces local computation and avoids the problem of dimension difference, it sacrifices accuracy and increases global computation by using incomplete information. To address the issue of dimensional disparity, this paper proposes the use of a vision transformer to collapse the velocity axis, which effectively transforms the range-azimuth-velocity data extracted from each layer into range-azimuth data for the decoder. As a result, the full radar data is utilized while also reducing computational requirements. The software for our pipeline is readily available on our website at https://github.com/heliluya/radar-target-recognition accessed on 14 November 2023. Next, we will provide detailed descriptions of the function and structure of each module in the network.

### 2.1. Input Data

The network is designed to process radar heatmaps with velocity information, as depicted in Figure 1. Therefore, the size of the input data is $1\times H_{0}\times W_{0}\times L_{0}$, which has only one channel. Moreover, $H_{0}$ represents the range of the range dimension; $W_{0}$ represents the range of the azimuth dimension; $L_{0}$ represents the range of the velocity dimension. It is essential to note that input data must be standardized before feeding into the network to enhance network stability and robustness. Unlike visible light images, where different cameras can have standardized parameters, different radars and environments can produce different echo intensities, leading to the need for distinct standard means and deviations for different radars or environments. Typically, standard means and variances are determined by the training set. The standardized operation is

(1) $$\mu =\frac{\sum_{n}^{N}\sum_{h}^{H_{0}}\sum_{w}^{W_{0}}\sum_{l}^{L_{0}}p_{n,h,w,l}}{N\times H_{0}\times W_{0}\times L_{0}},$$

and

(2) $$\sigma =\sqrt{\frac{\sum_{n}^{N}\sum_{h}^{H_{0}}\sum_{w}^{W_{0}}\sum_{l}^{L_{0}}{\left(p_{n,h,w,l}-\mu \right)}^{2}}{N\times H_{0}\times W_{0}\times L_{0}}},$$

where $p_{n,h,w,l}$ represents the intensity of the *h* point on the range axis, the *w* point on the azimuth axis, and the *l* point on the velocity axis in the *n*-th radar diagram.

### 2.2. Encoder

The main objective of the encoder is to extract features by various levels from input data, with each level representing radar targets with varying levels of detail. Since radar data involves three dimensions (range, azimuth, and velocity), it is necessary to use 3D operators to extract features during encoding. The encoder module comprises four layers, each of which contains two 3D Convolutional layers, a Dropout Operator, and a 3D Max Pooling Operator arranged sequentially. The output channel size of each layer corresponds to 64, 128, 256, and 512, respectively, from the low layer to the high layer. The 3D Convolutional layer uses a kernel size of 3×3×3, padding of 1, and stride of 1 to extract features, while the 3D Max Pooling Operator has a kernel size of 2×2×2, padding of 0, and stride of 2 to down-sample the spatial dimensions. If the input of layer *i* of the encoder is characteristic $F_{i}\in {ℜ}^{C_{i-1}\times H_{i-1}\times W_{i-1}\times L_{i-1}}$, where $C_{0}$ is 1, the output of layer *i* is calculated as

(3) $$O_{i}=MaxPool(Dropout(3DConv_{i}(3DConv_{i}(F_{i})))),$$

where $3DConv_{i}$ is the 3D Convolution Operator. The output of this module is a 512×H4×W4×L4 tensor.

### 2.3. Decoder

The role of the decoder is to generate semantic segmentation outcomes of varying levels layer by layer based on the radar target representation. Each layer’s output is a sum of the previous layer’s result and the current layer’s result, leading to the final semantic segmentation output. The decoder processes a 2D tensor with a size of $1024\times H_{4}\times W_{4}$, obtained by Channel×Range×Azimuth. The data dimensions of the encoder and decoder in the segmentation network proposed in this paper are different, unlike the U-Net. The encoder data is 3D, while the decoder data is 2D. During the encoder phase, the convolution of the velocity dimension enables better capturing of target features. However, in the output segmentation result, there is no need to separate the velocity dimension since it belongs to the same target. Consequently, in the decoder phase, generating the semantic segmentation result of the velocity dimension is unnecessary. This absence of the velocity dimension makes the data in the decoder two-dimensional. The decoder, like the encoder, comprises four layers, with the first operation of each layer involving upsampling in every dimension by a scale factor of 2. The second operation involves stitching the upsampled outcomes with the intermediate features of the corresponding layers’ number in the encoder. The third operator is two 2D Convs., with a kernel size of 2×2, padding of 1, and stride of 1. The fourth operator is Dropout. The output channel size of each layer corresponds to 512, 256, 128, and 64, respectively, from the high layer to the low layer. Assuming the decoder’s layer *i* input is feature $G_{i}\in {ℜ}^{(2\times C_{i-1})\times H_{i-1}\times W_{i-1}}$, the output of layer *i* is calculated as follows:

(4) $$D_{i}=Dropout(2DConv_{i}(2DConv_{i}(UpSample(G_{i})+Dropout(3DConv_{i}(3DConv_{i}(F_{i})))))),$$

where the $2DConv_{i}$ is the 2D Convolution Operator. The output of this module is a 512×H4×W4×2 tensor.

### 2.4. Connection between Encoder and Decoder

The above results show that the encoder produces a tensor of size $512\;\times \;H_{4}\times W_{4}\times L4$, whereas the decoder requires an input tensor of size $1024\;\times \;H_{4}\times W_{4}$. Therefore, certain operations are needed to transform the channel and dimensions between the encoder and decoder. This requires the use of dimension collapse. The connection between the encoder and decoder is located in the deep layers of the neural network, and the features in this connection have been downsampled multiple times. As a result, the number of velocity axis parameters that need to be collapsed is small. To achieve dimension transformation, the velocity dimension of the encoder can be spliced together, and the channel size can be changed using convolution. The connection between the encoder and decoder comprises two 3D Conv. operations, a concatenation operator, and a 2D Conv. operation. The 3D Conv. operations are used to extract features from the data after downsampling, with an output channel size of 1024, kernel size of 3×3×3, padding of 1, and stride of 1. The 2D Conv. operation changes the number of channels and performs feature transformation, with an output channel size of 1024, kernel size of 1×1, padding of 0, and stride of 1. In summary, the connection module between the encoder and decoder can be described as follows:

(5) $$G_{0}=2DConv(Concat(3DConv(3DConv(O_{4})))),$$

where *Concat* represents the splicing of the velocity dimension along the channel dimension.

### 2.5. Skip Connection between Encoder and Decoder

The distinguishing feature of the U-Net is the presence of skip connections between the encoder and decoder, which allows the model to generate results using both deep semantic information and shallow positional information. However, the dimensions of the features extracted from each layer of the encoder and decoder in the proposed model in this paper are different and cannot be directly fused through the skip connection. Therefore, it is necessary to collapse the velocity dimension of the encoder’s feature before passing it through the skip connection, so that its dimension matches that of the decoder. Velocity dimension collapse essentially involves extracting global features from the velocity dimension. Convolution operation can only extract local features, while fully connected layers for extracting global features significantly increase the number of operations. Global maximum pooling or global average pooling can also extract global features, but these operators are rather crude. The module divides the input into multiple blocks along the range and azimuth dimensions, and each block is further divided into multiple patches along the velocity dimension. Each patch consists of channel information for specific range, azimuth, and velocity. Subsequently, patches with different velocity at the same range and azimuth are simultaneously input into the Transformer module to fuse the velocity information. Global mean pooling is operated on the fusion results to obtain velocity fusion information at specific range and azimuth. Finally, the velocity fusion information from different range and azimuth will be concatenated as channels to obtain data in the form of range-azimuth-channel. Compared to Convolutional layers, pooling operators, and fully connected layers, transformers can extract global features more effectively at a lower computational cost. Therefore, the vision transformer is introduced into the skip connection between the encoder and decoder to extract global features and collapse the velocity dimension of the encoder [24]. The feature tensor size extracted from each layer of the encoder is B×C×H×W×L, where B represents the batch size. To match dimensions, the input of the transformer w is given as:

(6) $$w=flat\left(permute\left(v\right)\right).$$

The size of w is $\left(B\times H\times W\right)\times L\times C$. The role of permute() is to rearrange the integration dimensions. Before W is input into the transformer, a trainable location embedding is added. The size of the positional embedding is the same as that of the transformer. The purpose of the location embedding is to enable the network to adapt to inputs of different lengths. The calculation flow of W in the transformer module is as follows:

(7) $$\begin{array}{l} z_{0}=w+w_{pos},\\ z_{l}^{\prime }=MSA(LN(z_{l-1}))+z_{l-1},l=1\ldots L,\\ z_{l}=MLP(LN(z_{l}^{\prime }))+z_{l}^{\prime },l=1\ldots L,\\ y=LN(z_{L}), \end{array}$$

where the MSA() represents the Multi-headed Self-attention operator, MLP() represents the full connection layer, LN() represents the layer normalization, and L is the number of encoder layers in the transformer. After the transformer operation, each data point contains global information pertaining to the velocity axis of the data y. The feature of the collapsed velocity axis can be obtained by performing global pooling on the velocity axis of data y. The output out of the skip connection is obtained by reshaping the features after the collapsed velocity axis, and its size is B×C×H×W. In summary, the output out of the skip connection can be calculated as:

(8) out=Reshape(MeanPool(y)).

### 2.6. Other Modules of the Network

It is important to recognize that the channel size of the data produced by the decoder may not match the number of classification categories. In order to ensure that the predicted results of semantic segmentation align with the desired number of classifications, a Segmentation Head is necessary following the decoder. This component is implemented as a Convolutional layer with a kernel size of 1×1, padding is 0, and a stride of 1. The Stochastic Gradient Descent Optimizer is utilized in the network, and the loss function is defined as follows:

(9) $$L=L_{ce}+L_{dice},$$

where $L_{ce}$ denotes the Cross-Entropy Loss and $L_{dice}$ denotes the Dice Loss.

## 3. Experiment

### 3.1. Data

In order to validate the effectiveness of 3DCUNet, we utilized the open-source dataset SCORP [25]. The data was collected using a mobile device that is equipped with a side view radar and a camera, with the objective of detecting open and removable space within a parking lot. The SCORP dataset was created by using a linear frequency modulation continuous wave radar operating at 76 GHz frequency, in Multiple Input Multiple Output Mode (MIMO). The radar system operates through the use of 2 Tx elements, which transmit sequentially, and 4 Rx elements that receive coherently in the Time Division Multiplexing MIMO mode, resulting in 8 virtual channels for radar information. For more information regarding the radar configurations used in this study, please refer to [25].

The SCORP dataset comprises two types of output data, radar and camera data. The radar data is obtained through analog-to-digital conversion and is processed using Fast Fourier Transform along with Samples, Chirps, and Antenna dimensions to produce Range-Doppler-Azimuth representation (RDA), which is used as the input for this research. On the other hand, the camera output data is used to annotate the radar data, which is divided into two categories: removable space and immovable space. The dataset comprises 3913 frames collected in 11 sequences, with each RDA frame consisting of a 256×256×64 matrix representing the amplitude of each position and velocity. However, using the large 256×256×64 matrix as the network input consumes significant computing resources. To overcome this, the matrix is resized using Bilinear Interpolation to a 128×128×32 matrix. The testing set comprises a 10% split of the SCORP dataset after shuffling, which was also used as the training set.

### 3.2. Results

The training process is carried out using PyTorch-1.12.1 [26] on a 3.50 GHz CPU and an NVIDIA GTX3080 GPU with 12 GB memory. Different dropout probabilities are set for different convolution layers: 0.1 for the convolution layer with output channel 64 or 128, and 0.2 for the convolution layer with output channel 256 or 512. The batch sizes for all experiments are set to 16, and the networks are trained until the 10th epoch while adopting the Early Stop Method. During training, the optimizer learning rate is set to 0.1, momentum is set to 0.9, and L2 regularization is adopted with a weight decay of 0.0001. To save computing resources, 16-bit Floating-Point Numbers are used for training. The network performance is evaluated using the Mean Intersection over Union (MIoU) method, which is defined as

(10) $$MIoU=\frac{1}{k}\sum_{i=0}^{k}\frac{p_{ii}}{\sum_{j=0}^{k}p_{ij}+\sum_{j=0}^{k}p_{ji}-p_{ii}}.$$

Equation (10) in this paper uses k to denote the number of categories, which is 2 in this case. The variables $p_{ii}$, $p_{ij}$, and $p_{ji}$ represent true positive, false positive, and false negative, respectively. To ensure the accuracy of the results, all outcomes undergo K-fold cross-validation.

The complexity of radar data poses a challenge for radar data processing algorithms, as they often target specific data formats, making it difficult to adapt them to fit the datasets used in this paper. Therefore, to evaluate the performance of the proposed network architecture, the U-Net is used as the baseline. However, the input of the U-Net only accepts two-dimensional data, which requires the Global Mean Pooling Operation to collapse the velocity dimension of the input 3D radar heatmaps to obtain 2D data. Subsequently, the 2D data is fed into the U-Net for radar target recognition. The second comparison experiment differs from the proposed network architecture in only one aspect: the way the velocity dimension is collapsed. Contrast test 2 stitches the velocity axis along the channel dimension and convolves to collapse the velocity dimension, without utilizing the vision transformer. The velocity dimension of contrast test 2 collapses in the same way as the connection between the encoder and decoder.

**Figure 3:**
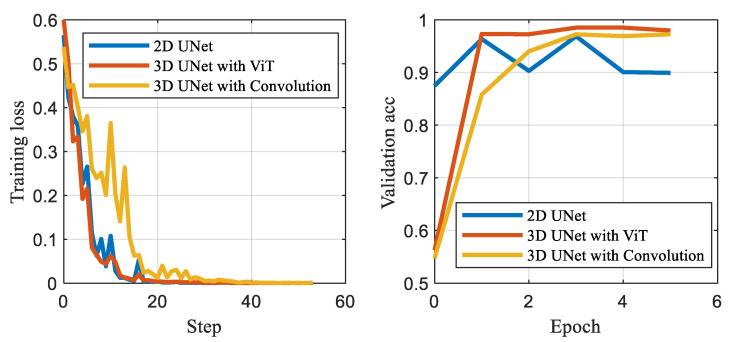
The curves of loss and accuracy under the different networks.

Table 1 presents the performance comparison between the 2D U-Net, the 3D U-Net that utilizes Convolutional collapse to reduce the velocity dimension, and the proposed 3D U-Net that adopts the Vision Transformer to collapse the velocity dimension. Results demonstrate that the 3D network outperforms the 2D radar segmentation network. Moreover, the metrics of the 3D U-Net with the Vision Transformer show slightly better performance in terms of MIoU than those of the 3D U-Net with convolutional collapse. The average loss and accuracy curve is shown in Figure 3, which shows a clear improvement in performance with 3D Collapse U-Net compared to other methods and the baseline model. Furthermore, by using the Vision Transformer, the proposed 3D U-Net achieves a 50% reduction in the number of parameters compared to the Convolutional collapse method.

## 4. Discussion

The experimental results clearly demonstrate that the 3D network outperforms the 2D radar segmentation network. This can be attributed to the 3D radar heatmap’s ability to provide more detailed information and more comprehensive characterization of the target compared to 2D radar data. Additionally, the global averaging operator in 2D networks fails to effectively collapse the velocity dimension, resulting in the loss of velocity characteristics. In contrast, experiments show that the 3D U-Net with a transformer module to collapse the velocity dimension outperforms a 3D U-Net with convolutional collapse. This can be explained by the global attention mechanisms in transformer modules, which enable more efficient feature collapse capabilities [24]. This is due to the ability of the vision transformer velocity dimension collapse module to acquire a similar capability to the full connected layer. Moreover, using a vision transformer to collapse the velocity dimension results in a smaller number of parameters compared to using Conv. This is because Conv. is equivalent to the full connection layer in the velocity dimension, leading to an increased number of parameters. While this shortcoming of Conv. is not obvious on RGB images, it becomes more apparent on velocity dimensions with larger dimensions. However, the connection of the attention mechanism is sparse, which leads to a significant decrease in the number of parameters.

This research introduces vision transformer technology into the field of radar target recognition, resulting in a significant improvement in the accuracy of radar target recognition. In addition, the improved performance also benefits from the selection of radar data types. The process of semantic segmentation for radar data differs from that of optical images. Unlike optical images, which have a unified format and rely on the algorithm or network models, radar data takes on various forms such as ADC, multidimensional heatmaps, and point cloud data [2,25]. These different data formats describe radar information at varying levels of granularity, making it essential to determine the appropriate input format before engaging in radar semantic segmentation. For example, Shahzad Ahmed and colleagues achieved gesture recognition by reorganizing ADC data as slow-fast time indexes [27]. Additionally, they optimized both the network and radar data while normalizing the mean and variance of the data to enhance the algorithm’s robustness. Although the ADC signal provides the most comprehensive information, it is also highly redundant due to the raw radar data being obtained from multiple quantitative samplings of the echo at the same orientation and angle. As a result, using the ADC signal directly for radar semantic segmentation requires significant computational resources. On the other hand, to further reduce computation, researchers use the Constant False Alarm Rate Method to process radar signals and obtain point cloud data for radar target detection. Ole Schumann et al. presented an end-to-end network to obtain semantic information for each point in the point cloud [28]. Their work divides static and dynamic objects into two separate branches, ultimately merging them into a point cloud. Experimental results show that the scheme is effective in automobile radar data processing. By using point cloud data, the radar semantics segmentation is completed with limited computing costs, which have been widely used in automatic driving [6]. However, it is worth noting that the point cloud data is obtained by utilizing the Constant False Alarm Detection Technique in the radar heatmap, which can result in only a few dozen points being generated. Consequently, the point cloud data can be quite sparse and may lack essential information, thus reducing its detection effectiveness. These heatmaps are generated from ADC using fast Fourier transform in different dimensions, resulting in a high-order tensor with abundant information that meets the requirements of semantic segmentation. There are also many researchers who have demonstrated and studied the results in this area [18,19,20]. A representative example is that Ignacio Roldan et al. addressed the issue of large data volume in radar heatmaps by using the Constant False Alarm Rate Method, which reduced the computational amount while maintaining high accuracy [29]. In contrast to the Constant False Alarm Rate Method that generates a point cloud, the constant false alarm algorithm presented in this paper produces a region as its output result.

In a word, by leveraging the more primitive and rich information contained in radar heatmaps, this approach offers a more comprehensive and accurate representation of the real situation. The application of radar heatmaps in industries such as mobile robotics, autonomous driving, and underground exploration is also promoted by this research.

At present, there is still room for further refinement in this research. The performance of the velocity collapse module and the radar semantic segmentation network needs to be tested in more diverse datasets and application scenarios. In addition, future research can focus on a more detailed analysis of the performance of the dimensional collapse module and further explore the possibilities of semantic segmentation based on radar heatmaps.

## 5. Conclusions

This paper proposes a novel network architecture for radar semantic segmentation, called 3D Collapse U-Net, which utilizes a three-dimensional radar heatmap that includes range, azimuth, and velocity information. The 3D Collapse U-Net is based on the popular UNet architecture and uses 3D convolution operators. It outperforms networks processing 2D data and using convolution operators to collapse the velocity dimension, with fewer parameters and better performance. The dimension collapse module is the core of the network, which leverages the global attention capability of the vision transformer to collapse the specified dimensions. This module is highly scalable and can be used to process other high-dimensional data. Its performance is better than that of dimensional collapse based on global mean pooling or convolution operators. The network’s performance is validated through experiments on semantic segmentation using the SCORP dataset. Further research can explore the applicability of the 3D Collapse U-Net architecture to other types of data and applications.

## Figures and Tables

**Table 1:** The performance of different networks. The sign ± indicates the results of K-fold cross-validation.

Method	Param (MB)	MIoU
2D UNet	62.07	96.86 ± 0.21
3D UNet with Convolution	210.57	97.25 ± 1.78
3D UNet with ViT	169.58	98.51 ± 0.81

## Data Availability

Publicly available datasets were analyzed in this study.
